# Unequal bonding in Ag–CuIn_3_Se_5_-based solid solutions responsible for reduction in lattice thermal conductivity and improvement in thermoelectric performance†

**DOI:** 10.1039/c8ra00316e

**Published:** 2018-03-05

**Authors:** Jiaolin Cui, Yufu Lu, Shaoping Chen, Xianglian Liu, Zhengliang Du

**Affiliations:** School of Materials & Engineering, Ningbo University of Technology Ningbo 315016 China cuijiaolin@163.com; Materials Science and Engineering College, Taiyuan University of Technology Taiyuan 030024 China

## Abstract

Owing to their unique crystal and band structures, in thermoelectrics increasing attention has recently been paid to compounds of the ternary I–III–VI chalcopyrite family. In this work, unequal bonding between cation and anion pairs in Cu_1−*y*_Ag_*y*_In_3_Se_4.9_Te_0.1_ solid solutions, which can be effectively used to disturb phonon transport, has been proposed. The unequal bonding, which is represented by the difference of bond lengths Δ*d*, Δ*d* = *d*_(Cu–Se)_ − *d*_(In–Se)_ and anion position displacement from its equilibrium position Δ*u* = *u* − 0.25, is created by the isoelectronic substitution of Ag for Cu. At *y* = 0.2 both the Δ*d* and Δ*u* values reach their maxima, resulting in a remarkable reduction in lattice thermal conductivity (*κ*_L_) and an improvement in TE performance. However, as the *y* value increase to 0.3 both Δ*d* and Δ*u* values decrease, causing the *κ*_L_ value to increase and the ZT value to decrease from 0.5 to 0.24 at 930 K. Accordingly, unequal bonding might be an alternative way to improve the TE performance of ternary chalcopyrites.

## Abstract 2

An unequal bonding in the CuIn_3_Se_5_ ternary chalcopyrite is responsible for the reduction in lattice thermal conductivity and improvement in thermoelectric performance.

## Graphical abstract

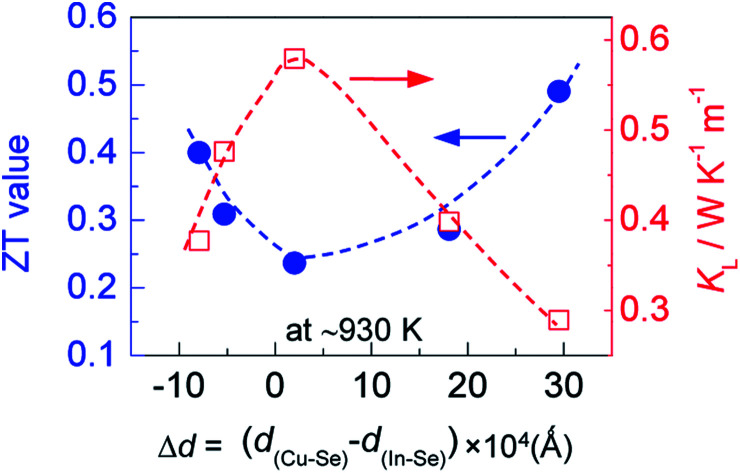

## 1. Introduction

Compounds of the ternary I–III–VI chalcopyrite family have been paid more and more attention in thermoelectrics (TE)^1–3^ since they have two types of inherent feature structures, which are namely, crystal structure defects and band splitting. The crystal structure defects mainly refer to the anion position displacement, tetragonal distortion and donor–acceptor defect pairs (DADPs) with Coulomb attraction between charged III_I_^2+^ and 2V_I_^−^.^4–6^ The tetragonal distortion can be visualized as the local anion displacement from its equilibrium position, *i.e.*, bond alteration between two cation–anion pairs (I–VI and III–VI). This alteration gives rise to a non-zero valence band split (*Δ*_CF_) along with the creation of the compression or tensile stress in the lattice. In this case, the anion position displacement *u*, 
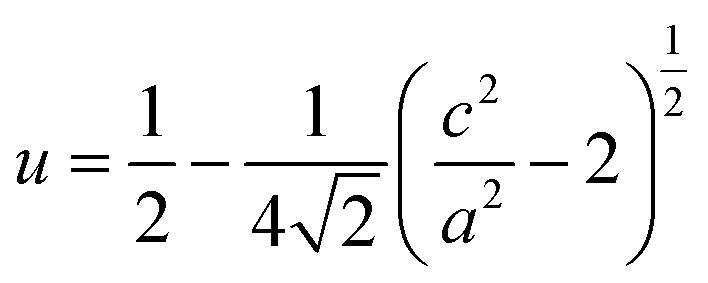
,^7,8^ does not equal to 0.25, or *η* = *c*/2*a* ≠ 1, where *a* and *c* are the lattice constants. Since the inherent feature structures are critical in engineering the TE performance of the chalcopyrites, many approaches have been conceived of, such as, the cation vacancy concentration or vacancy scattering engineering,^9–11^ carrier-magnetic moment interaction employment^12^ and nano-structure manipulation.^13,14^ In addition to the above-noted strategies, there is a unity-*η* rule proposed recently.^2,15^ This rule indicates that the power factors (PF) display peak values when *η* is around 1 or when crystal field splitting energy *Δ*_CF_ equals to zero. However, recent works reveal that for each ternary chalcopyrite with several space groups the best thermoelectric performance can only be achieved at a certain *u* value (*u* ≠ 0.25).^16,17^ Because of the Coulomb attraction between charged III_I_^2+^ and 2V_I_^−^, the DADPs formed in the chalcopyrites are electrically inactive and their mutual interaction leads to a partial annihilation on their corresponding acceptor and donor levels.^18^ Therefore, when *u* equals to 0.25 or *η* to 1, a cubic-like structure forms, and the bond charges on I–VI and III–VI become almost equal. Under such circumstances, both the carrier concentration and electrical conductivity degrade.^19^ Further, when foreign elements with different atomic radius and electronegativity are incorporated into either I or III site, the bond charges redistribute, and will thus generate unequal bond lengths. This change might directly distort crystal structure and disturbs phonon transport. For example, when an element Ag iso-electronically substitutes for Cu^20,21^ or In for Ga in CuGaTe_2_,^22,23^ the phonon scattering intensifies and lattice thermal conductivity reduces significantly. The other examples are the incorporation of Mn into Cu site in CuInSe_2_ (ref. 24) and Sb into Cr site in CuCr_2_S_4_,^25^ which expands the crystal structure and increases the electrical conductivity and Seebeck coefficient simultaneously, in addition to the disordering of lattice structure.^26^

In view of the above arguments, it is strongly necessary to clarify what role of the unequal bonding in ternary chalcopyrites plays in influencing the carrier and phonon transport?

Based on the previous investigations,^16^ in this work we have proposed an unequal bonding between Cu–Se and In–Se, which is inspired by the isoelectronic substitution Ag for Cu, in the Te–CuIn_3_Se_5_ system. The existence of unequal bonding environment reduces the lattice thermal conductivity and improves the TE performance.

## 2. Experimental

### 2.1 Sample preparation

The mixtures, according to the chemical formula Cu_1−*y*_Ag_*y*_In_3_Se_4.9_Te_0.1_ (*y* = 0.05, 0.1, 0.2 and 0.3) with the purity of above five elements more than 99.999%, were loaded into four different vacuum silica tubes and melted at 1273 K for 24 h, followed by a rapid cooling to room temperature (RT) in water. The solidified ingots were then pulverized and ball milled in stainless steel bowls containing benzinum at a rotation rate of 350 rpm for 5 h. The dried powders were sintered using spark plasma sintering apparatus (SPS-1030) under a pressure of 60 MPa and at the highest temperature of ∼950 K. The total sintering time is about 5 min. The densities (*d*) of the polished bulks, which have more than 95% theoretical density, were measured using Archimedes' method.

The final size of the samples used for electrical property measurements including Seebeck coefficient (*α*) and electrical conductivity (*σ*) is 3 mm in thickness and 2.5 mm × 12 mm in cross-section, cut from the sintered blocks (*ϕ* 20 mm × 2.5 mm), and that for thermal diffusivity (*κ*) measurements is *ϕ* 10 mm × 1.5 mm.

### 2.2 Physical property measurements

Both the Seebeck coefficient (*α*) and electrical conductivity (*σ*) were measured simultaneously under a helium atmosphere from RT to ∼930 K on a ULVAC ZEM-3 instrument system with an uncertainty of <6.0% for each. The thermal conductivities were calculated based on the equation *κ* = *dλC*_p_, where the thermal diffusivities *λ* were measured by TC-1200RH instrument under vacuum (uncertainty < 10.0%). Since the Debye temperature of CuIn_3_Se_5_ is about 260 K,^27^ it is reliable that the Dulong–Petit rule is used to estimate the heat capacities (*C*_p_) above RT. The three physical parameters (*α*, *σ*, *κ*) were finalized by taking the average values of several samples tested by the same method. The lattice contribution (*κ*_L_) was the total *κ* minus the electronic contribution (*κ*_e_). Here *κ*_e_ is expressed by Wiedemann–Franz (W–F) relation, *κ*_e_ = *L*_0_*σT*, where *L*_0_ is the Lorenz number, estimated to be 1.5 × 10^−8^ W Ω K^−2^ for not fully degenerate environment of semiconductors.^15^ The TE figure of merits (ZTs) were calculated from the above three parameters according to the equation, ZT = *α*^2^*σ*/*κ*, with the total uncertainty of about 18%. Hall coefficients (*R*_H_) were measured by using a four-probe configuration in a system (PPMS, Model-9) with a magnetic field up to ±2 T. The Hall mobility (*μ*) and carrier concentration (*n*_H_) were subsequently calculated according to the relations *μ* = |*R*_H_|*σ* and *n*_H_ = −1/(*eR*_H_) respectively, where *e* is the electron charge.

### 2.3 Chemical compositions and structural analyses

The chemical compositions of the sample (*y* = 0.2) were checked using an electron probe micro-analyzer (EPMA) (S-4800, Hitachi, Japan) with an accuracy of >97%.

The XRD patterns were collected by powder X-ray diffractometer (D8 Advance) operating at 50 kV and 40 mA at Cu Kα radiation (*λ* = 0.15406 nm) and a scan rate of 4° min^−1^ in the range from 10° to 110°. The lattice constants *a* and *c* were obtained from the refinement of the X-ray patterns using Jade software.

Raman spectra of four Cu_1−*y*_Ag_*y*_In_3_Se_4.9_Te_0.1_ powders (*y* = 0.05, 0.1, 0.2 and 0.3) were recorded at 300 K from 50 to 4000 cm^−1^ at a resolution of 0.6 cm^−1^ using an Invia-Reflex Raman spectrometer with Nd:YAG laser source (*l* = 532.0 nm), and that of the sample CuIn_3_Se_5_ was presented for comparison. A 50× objective lens was employed *via* a confocal geometry to transmit the incident laser beam and collect the scattered radiation. Laser intensity was mandatorily attenuated to prevent the samples from decomposing.

## 3. Results and discussions

### 3.1 Composition analyses and XRD

The mapping pictures of five elements Cu, In, Se, Te and Ag and EDAX spectrum for the sample at *y* = 0.2, under scanning electron microscopy (S-4800, Hitachi, Japan), are shown in Fig. S1.† Generally, the elements are uniformly distributed with a subtle segregation, as shown in Fig. S1a–e.† The chemical compositions taken from a mapping are presented in Table S1,† where the number of moles of Se is normalized to 4.9. The relative molars of Cu, In, Se, Te and Ag identified are close to those of nominal compositions, only a little deficiency in Te. Fig. S1f† is the EDAX spectrum. These results suggest that the compositions are almost as intended as the final samples. Besides, an average grain size of the sample at *y* = 0.2 is determined to be 10–15 μm, observed by using back-scattered electron image (BSEI) (Fig. S1g†).

**Fig. 1:**
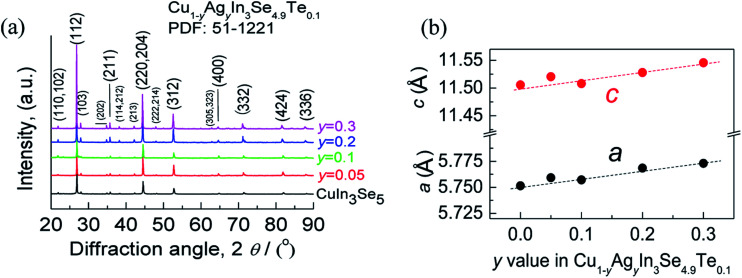
(a) XRD patterns of the Cu_1−*y*_Ag_*y*_In_3_Se_4.9_Te_0.1_ powders; (b) lattice constants *a* and *c* values.

**Fig. 2:**
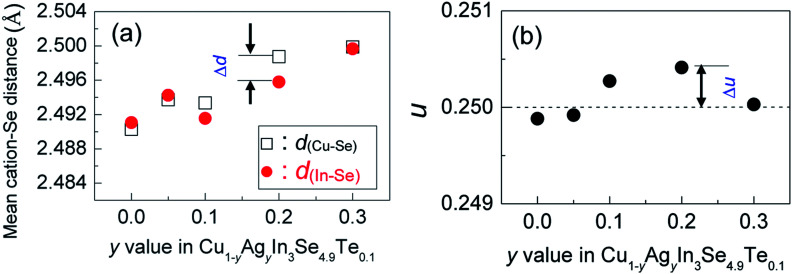
(a) Cation–anion distances *d*_(Cu–Se)_ and *d*_(In–Se)_, here the Δ*d* represents the difference of the cation–anion distances; (b) the defect parameter *u* values as a function of Ag content (*y*) in Cu_1−*y*_Ag_*y*_In_3_Se_4.9_Te_0.1_, and Δ*u* = *u* − 0.25 stands for the deviation of the anion from its equilibrium position.

The X-ray diffraction patterns of the powders Cu_1−*y*_Ag_*y*_In_3_Se_4.9_Te_0.1_ (*y* = 0.05, 0.1, 0.2 and 0.3) at RT are shown in Fig. 1a, where all peak positions are observed the same as those of stoichiometric CuIn_3_Se_5_ (PDF: 51-1221) without any visible impurity phases identified. The result thus indicates that the synthesized samples are crystallized in a single phase. The lattice constants *a* and *c* as a function of *y* value, as shown in Fig. 1b, ranging from 5.75 to 5.77 Å in *a* and 11.50 to 11.55 Å in *c*, increase linearly with *y* value increasing. This indicates that the incorporation of Ag into the crystal lattice yields the dilation of the crystal structure as a result of the creation of local tensile stress caused by the larger atomic size of Ag than that of Cu. Besides, the obtained *a* value in CuIn_3_Se_5_ is comparable to those reported,^28–31^ but *c* value is a little lower.^28,29^ If more Ag is added, extra Ag might be energetically favorable to In site to release the internal stress. Such an reoccupation was also observed in the Cu_2_Zn_1−*x*_Fe_*x*_GeSe_4_ system,^32,33^ where Zn is forced to move to 4d-site from its original 2a-site as Fe content increases to *x* = 0.3–0.7. Because of the reoccupation of Ag, an redistribution of bond charges occurs between Cu–Se and In–Se. In this regard, it is possible that a new bonding environment will be created. This argument can be verified by the variations of the differences (Δ*d*) and Δ*u*, suggested in Jaffe and Zunger proposal.^4,5^ As *y* value increases, both the Δ*d* and Δ*u* increase and reach the maximum until at *y* = 0.2, and then reset somewhere close to zero simultaneously at *y* = 0.3, as shown in Fig. 2.

### 3.2 Transport properties

**Fig. 3:**
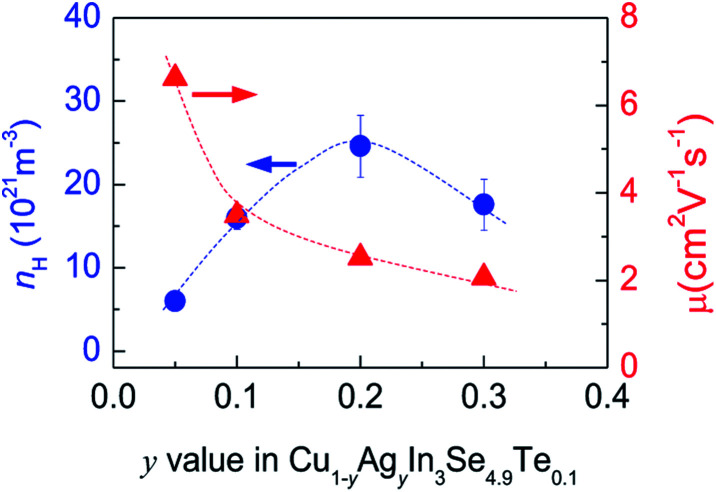
Measured Hall carrier concentration (*n*_H_) and mobility (*μ*) against *y* value in Cu_1−*y*_Ag_*y*_n_3_Se_4.9_Te_0.1_.

Many investigations reveal that in ternary chalcopyrites the p–d-hybridization between Cu 3d and anion p-orbitals governs the bandgap,^5,31,34^ thus influencing the net charge of electrons. The magnitude of Δ*d* value is, nonetheless, obviously related to the p–d-hybridization. In order to probe into the effect of Δ*d* value on the transport properties, the Hall coefficients (*R*_H_) at RT were measured. The Hall carrier concentration (*n*_H_) and mobility (*μ*) are calculated and presented in Fig. 3. It was observed that the *n*_H_ value enhances at first from 6.01 × 10^21^ cm^−3^ to 24.6 × 10^21^ cm^−3^ as *y* value increases from 0 to 0.2, and then reduces from 24.6 × 10^21^ cm^−3^ (*y* = 0.2) to 17.6 × 10^21^ cm^−3^ (*y* = 0.3). The reduction of *n*_H_ value at *y* > 0.2 seems to coincide with the equalization of the bonding or the decrease in Δ*d* value (Fig. 2a). Although the *n*_H_ values (6.01–24.6 × 10^21^ cm^−3^) in the present material system is much smaller than the optimal one (10^25^ to 10^26^ m^−3^) in thermoelectrics,^35^ they are comparable to (even larger than) those reported.^30,36^ The mobility (*μ*) monotonously decreases from 6.62 cm^2^ V^−1^ s^−1^ to 2.06 cm^2^ V^−1^ s^−1^ as *y* increases from 0.05 to 0.3 (Fig. 3).

### 3.3 TE performance

**Fig. 4:**
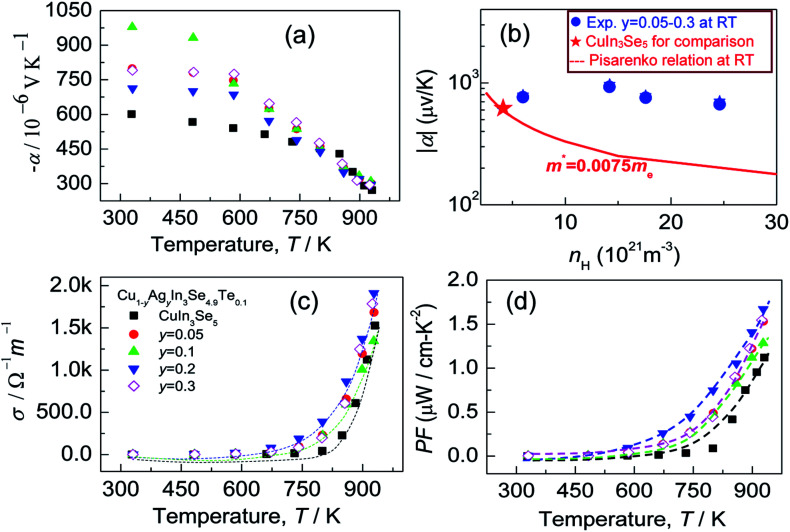
(a) Seebeck coefficients (*α*) of the compounds Cu_1−*y*_Ag_*y*_In_3_Se_4.9_Te_0.1_ (*y* = 0.05, 0.1, 0.2 and 0.3), and those of CuIn_3_Se_5_ are presented for comparison; (b) the experimentally determined |*α*| against *n*_H_ value for *y* = 0.05–0.3 at RT, labeled by 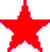, 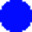. The red solid line represents the Pisarenko relation at RT; (c) electrical conductivities (*σ*); (d) power factor PF, PF = *α*^2^*σ*, as a function of temperature.

Since the carrier concentration and mobility have profound impacts on the electrical properties, we have therefore evaluated both the Seebeck coefficient (*α*) and electrical conductivity (*σ*) between RT to ∼930 K. The electrical properties of the pristine CuIn_3_Se_5_ are provided for comparison. It was observed that Seebeck coefficients are negative in the whole temperature range, as shown in Fig. 4a, which indicates a n-type semiconducting behavior of the materials. Besides, the absolute |*α*| value below ∼800 K increases and peaks when Ag content (*y* value) increases to *y* = 0.1 before decreasing. Above 800 K, the Seebeck coefficient gradually converges, and at ∼930 K the |*α*| value at *y* = 0.3 is 295.8 (μV K^−1^), only lower by about 8% compared to that of CuIn_3_Se_5_. Assuming that the Pisarenko relation below,^35^1
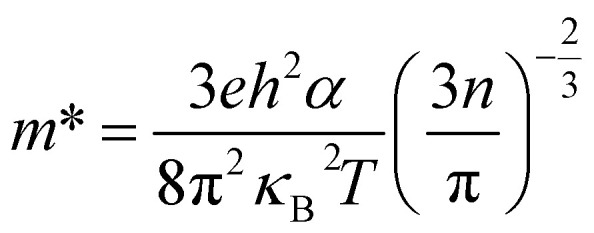
allows a proper depiction of the electron concentration-dependent Seebeck coefficients, the controlled Ag-incorporated samples give relatively higher Seebeck coefficients as compared to the Pisarenko plot, as indicated in Fig. 4b in red line. This Pisarenko relation corresponds to the m* value (0.0075*m*_e_) of CuIn_3_Se_5_, which is comparable to 0.006*m*_e_ in CuIn_3_Se_5_ thin film,^36^ suggesting that the effective mass of Ag-incorporated materials are larger than that of CuIn_3_Se_5_. Fig. 4c represents the electrical conductivity (*σ*) against temperature. As *y* value increases the electrical conductivity increases from 1.68 × 10^3^ Ω^−1^ m^−1^ (*y* = 0.05) to 1.91 × 10^3^ Ω^−1^ m^−1^ (*y* = 0.2) at ∼930 K, and at *y* = 0.3*σ* value decreases to 1.78 × 10^3^ Ω^−1^ m^−1^. With the Seebeck coefficients and electrical conductivities measured, we are able to attain the power factor (PF = *α*^2^*σ*), which reaches the highest (1.67 μW cm^−1^ K^−2^) at *y* = 0.2 and 930 K, as indicated in Fig. 4d.

**Fig. 5:**
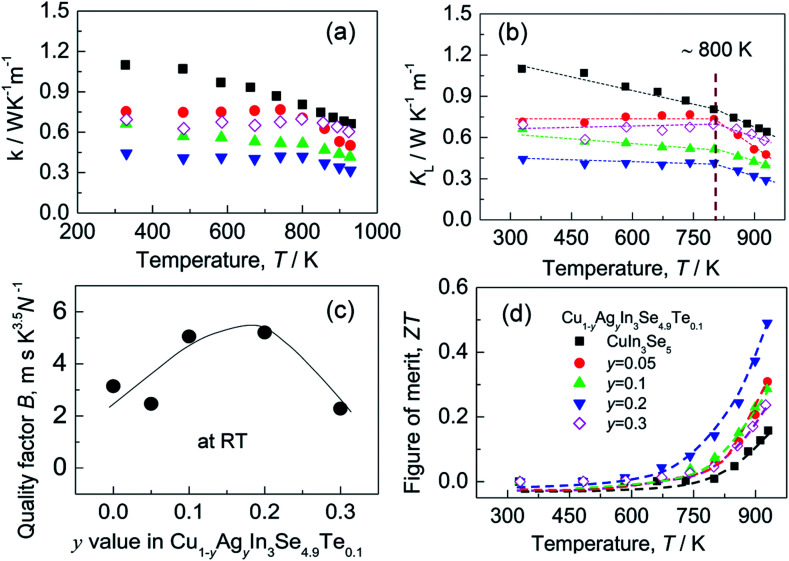
(a) Total thermal conductivities (*κ*); (b) lattice part (*κ*_L_); (c) the quality factor *B* values as a function of Ag content (*y* value); (d) TE figure of merit (ZT).

Although the electrical conductivity varies slightly with Ag content, the total thermal conductivity (*κ*) reduces significantly as Ag content increases, except for the sample at *y* = 0.3 (see Fig. 5a). The resemblance between lattice contribution (*κ*_L_) and total *κ* (Fig. 5b) suggests that the phonon transport dominates the heat carrying. It is noted that the temperature dependence of *κ*_L_ for Ag-incorporated samples can distinctly be divided into two linear parts, with a dividing point around 800 K. Above 800 K the *κ*_L_ values reduce more rapidly with temperature increasing, which indicates that the basic phonon scattering mechanism alters at high temperatures. The possible explanation for this phenomenon is an increase in phonon scattering at distorted lattices by an unequal bonding upon Ag incorporation, since the phonon scatterings in point defects and grain boundaries in this material system do not greatly impact the lattice thermal conductivity.^37^Fig. 5c displays a quality factor *B* (*B* = *μ*_H_(m*/*m*_e_)^3/2^*T*^5/2^/*κ*_L_)^15^ at RT. Generally, the *B* value increases with Ag content increasing, until it starts to decrease at *y* = ∼0.2. Combined with the three physical properties (*α*, *σ* and *κ*), we attain the TE figure of merit (ZT), as shown in Fig. 5d. Similarly, the ZT value reaches the highest (ZT = 0.5) at *y* = 0.2 and 930 K, ∼25% higher compared to ZT = 0.4 at *y* = 0.^16^ At *y* = 0.3, the ZT value decreases to 0.24. Therefore, Cu_0.8_Ag_0.2_In_3_Se_4.9_Te_0.1_ (*y* = 0.2) is the critical composition in this material system.

Upon limited Ag substitution for Cu in Cu_1−*y*_Ag_*y*_In_3_Se_4.9_Te_0.1_, Ag is energetically favorable to the Cu site. Under such a circumstance, the anion position has to be shifted by repelling the anion Se in order to equalize the occurring forces^5,38^ and thus elongate the bond length of Cu–Se (Fig. 2a). This change of bonding environment favors the overlap of orbitals and increases both the carrier concentration and effective mass (Fig. 3 and 4b), as was the cases in the solid solutions Cu_2_ZnGeS_4−*x*_Se_*x*_ (ref. 39–41) or Cu_2−*x*_Se.^42^

The existence of the repelling force upon Ag incorporation can be supported by the analyzed Raman spectra (shown in Fig. S2†), where the spectrum of CuIn_3_Se_5_ is presented for comparison. In the Raman spectra the intense A1 phonon signal at 152 cm^−1^ appears for all configurations,^43^ and the peak positions at 152 cm^−1^ and 177 cm^−1^*etc.* shift toward low-frequency side (red shift) as Ag content increases. This verifies the existence of the tensile stress caused by the changes of bonding environment.

Although the bonding environment change makes a limited improvement in electrical conductivity, the existence of the Cu–Se and In–Se bond networks, if only weakly disturbed, would change the phonon transport,^32,44^ thus reducing the lattice thermal conductivity. That is why we have observed the reduced lattice part *κ*_L_ and enhanced ZT value simultaneously when the difference of the bond lengths increases.

**Fig. 6:**
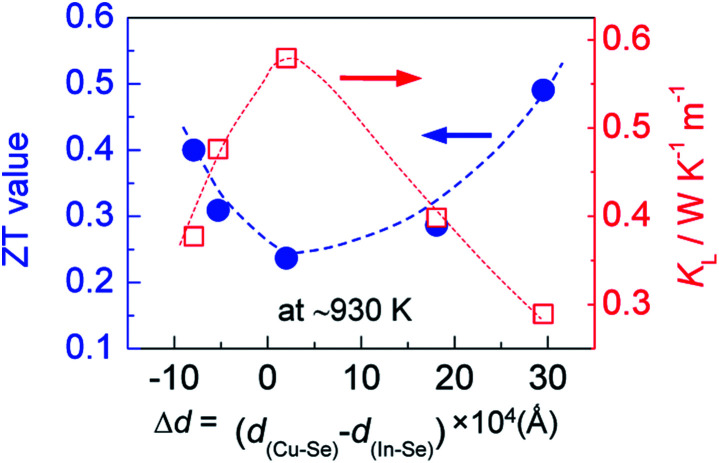
Relation of the ZT value at ∼930 K with the bonding length difference (Δ*d*), Δ*d* = *d*_(Cu–Se)_ − *d*_(In–Se)_, between cation and anion.

However, when more Ag is incorporated into the materials (for example, *y* = 0.3 at present material system), extra Ag atoms are forced to shift to the In site rather than Cu site, to release the internal stress and stabilize the structure. In this case, a more perfect tetrahedral angle of M–Se–M (M: metals) are gradually restored, and the bond lengths have to be adjusted and tends to become equal (Fig. 2c). In this case, the p–d hybridization between the metal d- and anion p-orbitals weakens,^39^ which leads to a degradation of the electrical conductivity (Fig. 4c) and enhances the lattice part *κ*_L_ (Fig. 5b). The *κ*_L_ and ZT values at ∼930 K as a function of bond length difference (Δ*d*), Δ*d* = *d*_(Cu–Se)_ − *d*_(In–Se)_, are summarized in Fig. 6, where it was observed that the larger the |Δ*d*| value, the lower the lattice part *κ*_L_ and higher the ZT value. This clearly indicates that an unequal bonding in Ag–CuIn_3_Se_5_-based solid solutions is responsible for the reduction in lattice thermal conductivity and improvement of thermoelectric performance.

## 4. Conclusions

Solid solutions Cu_1−*y*_Ag_*y*_In_3_Se_4.9_Te_0.1_ (*y* = 0.05–0.3) have been prepared, and an unequal bonding has been proposed through Ag isoelectronic substitution for Cu. This unequal bonding, which is represented by the difference of bond lengths Δ*d*, Δ*d* = *d*_(Cu–Se)_ − *d*_(In–Se)_ and anion position displacement from its equilibrium position Δ*u*, can be effectively used to disturb the phonon transport, when Δ*d* and Δ*u* reach the maximum at *y* = 0.2. Therefore, it reduces the lattice thermal conductivity (*κ*_L_) and improves the TE performance. However, as *y* value increase to 0.3 the bonding environment between cation and anion pairs tends to equalize in order to release the internal stress and stabilize the structure. As a consequence, the *κ*_L_ value enhances and ZT value decreases from 0.5 to 0.24 at ∼930 K.

## Conflicts of interest

There are no conflicts to declare.

## Supplementary Material

RA-008-C8RA00316E-s001
