# Supplementary material for: Unequal bonding in Ag–CuIn3Se5-based solid solutions responsible for reduction in lattice thermal conductivity and improvement in thermoelectric performance
Source: RSC Adv. 2018 Mar 5;8(17):9574–9. doi: 10.1039/c8ra00316e (PMC9078635; doi:10.1039/c8ra00316e)
Supplement: RA-008-C8RA00316E-s001 [file RA-008-C8RA00316E-s001.pdf]

Electronic Supplementary Material (ESI) for RSC Advances.
This journal is © The Royal Society of Chemistry 2018

Supporting information

Unequal bonding in Ag-$CuIn_3Se_5$-based solid solutions responsible for reduction in lattice thermal conductivity and improvement in thermoelectric performance

Jiaolin Cui,[a*] Yufu Lu,[a,b] Shaoping Chen,[b] Xianglian Liu,[a] Zhengliang Du[a]

Jiaolin Cui, Xianglian Liu, Zhengliang Du: School of Materials & Chemical Engineering, Ningbo University of Technology, Ningbo 315016, China

Yufu Lu, Shaoping Chen: Materials Science and Engineering College, Taiyuan University of Technology, Taiyuan, 030024, China.

Corresponding author's contact information:

Jiaolin Cui:

School of Materials and Chemical Engineering, Ningbo University of Technology, Ningbo 315211, China

E-mail: cuijiaolin@163.com

Tel: 86-574-87080504

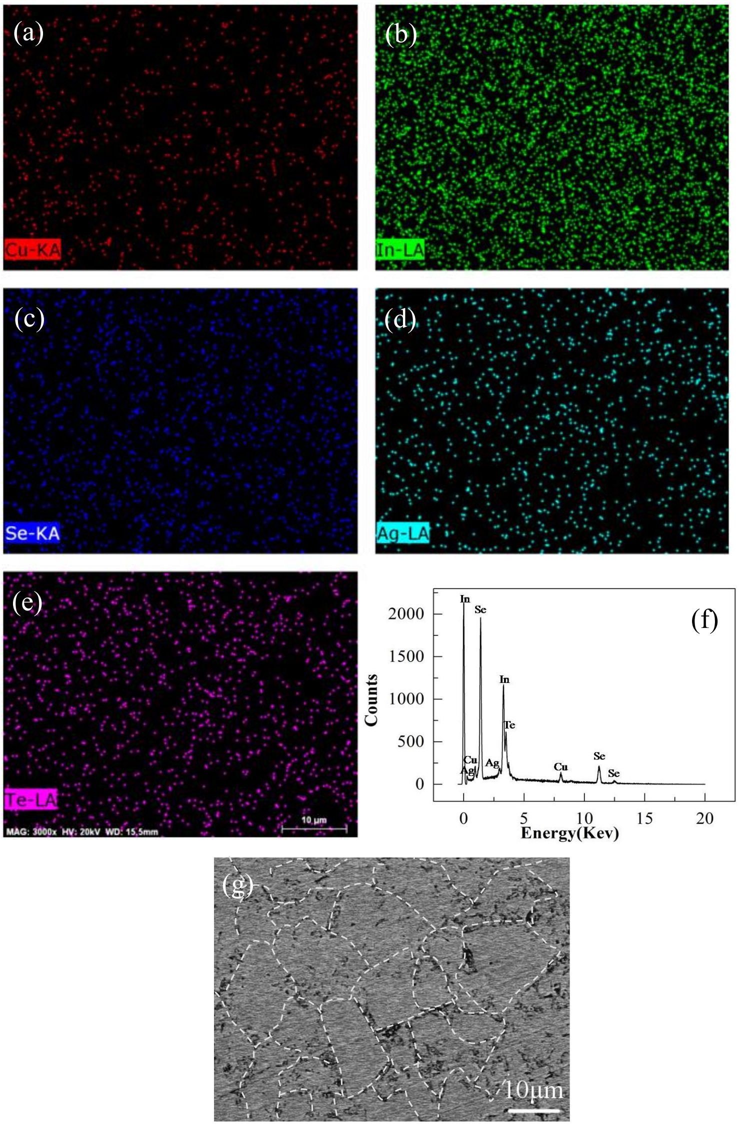

Fig.S1 (a-e) EPMA mapping of five elements, Cu, In, Se, Ag and Te on polished $Cu_{0.8}Ag_{0.2}In_3Se_{4.9}Te_{0.1}$ surface; (f) A SEM image, where an average grain size of 10~15 μm was observed; (g) EDAX analysis of the chemical compositions.

Table S1 The chemical compositions of the sample $Cu_{0.8}Ag_{0.2}In_3Se_{4.9}Te_{0.1}$ determined using EDAX.

Composition	Cu	In	Se	Te	Ag
$Cu_{0.8}Ag_{0.2}In_3Se_{4.9}Te_{0.1}$	0.81	2.95	4.90	0.06	0.17

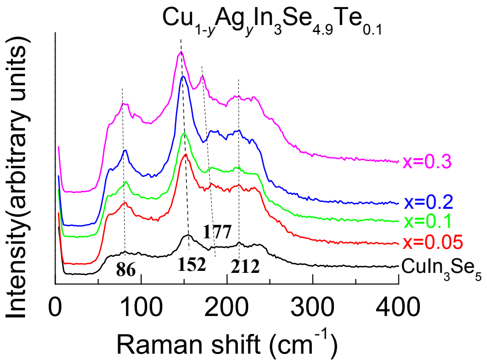

Fig. S2 Raman spectra of $Cu_{1-y}Ag_yIn_3Se_{4.9}Te_{0.1}$ (y=0.05, 0.1, 0.2 and 0.3) samples. The Raman spectrum of $CuIn_3Se_5$ is presented for comparison.
